# Remote sensing reveals Antarctic green snow algae as important terrestrial carbon sink

**DOI:** 10.1038/s41467-020-16018-w

**Published:** 2020-05-20

**Authors:** Andrew Gray, Monika Krolikowski, Peter Fretwell, Peter Convey, Lloyd S. Peck, Monika Mendelova, Alison G. Smith, Matthew P. Davey

**Affiliations:** 1 Department of Plant Sciences, University of Cambridge, Downing Street, Cambridge, CB2 3EA UK; 2 NERC Field Spectroscopy Facility, Edinburgh, EH3 9FE UK; 3 British Antarctic Survey, NERC, Madingley Road, Cambridge, CB3 0ET UK; 4 University of Edinburgh, School of GeoSciences, Edinburgh, EH8 9XP UK

**Keywords:** Plant sciences, Carbon cycle, Cryospheric science, Microbial ecology

## Abstract

We present the first estimate of green snow algae community biomass and distribution along the Antarctic Peninsula. Sentinel 2 imagery supported by two field campaigns revealed 1679 snow algae blooms, seasonally covering 1.95 × 10^6^ m^2^ and equating to 1.3 × 10^3^ tonnes total dry biomass. Ecosystem range is limited to areas with average positive summer temperatures, and distribution strongly influenced by marine nutrient inputs, with 60% of blooms less than 5 km from a penguin colony. A warming Antarctica may lose a majority of the 62% of blooms occupying small, low-lying islands with no high ground for range expansion. However, bloom area and elevation were observed to increase at lower latitudes, suggesting that parallel expansion of bloom area on larger landmasses, close to bird or seal colonies, is likely. This increase is predicted to outweigh biomass lost from small islands, resulting in a net increase in snow algae extent and biomass as the Peninsula warms.

## Abstract (web summary)

Snow algae bloom along the coast of Antarctica and are likely to be biogeochemically important. Here, the authors produced the first map of such blooms, show that they are driven by warmer temperatures and proximity to birds and mammals, and are likely to increase given projected climate changes.

## Introduction

**Fig. 1:**
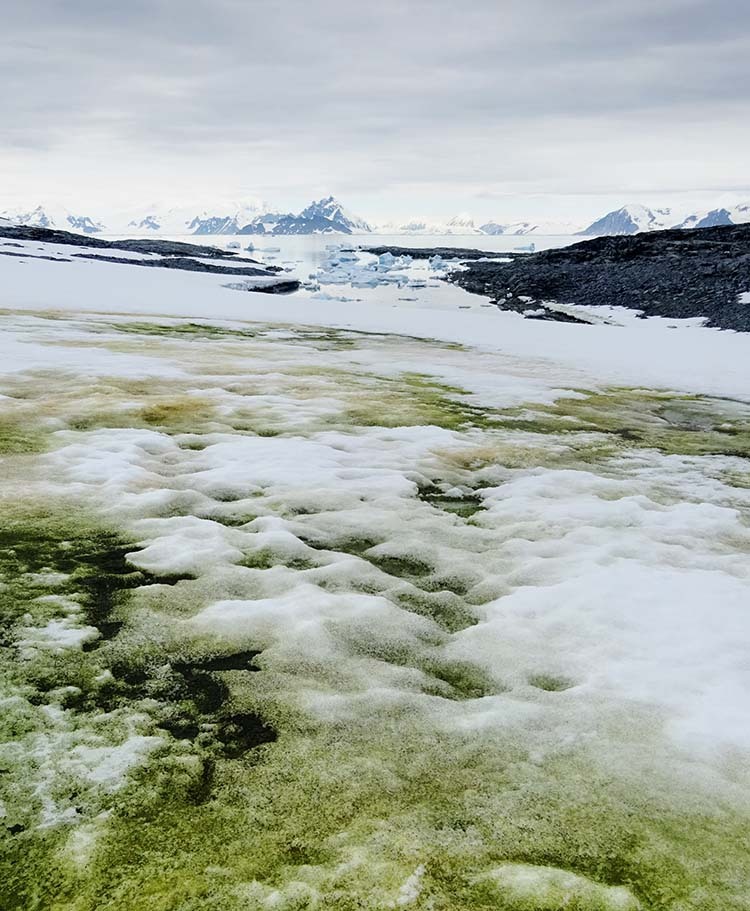
Green snow algae. A photograph showing a snow algae bloom dominated by green algae starting to melt out from beneath seasonal snow cover to sit exposed upon underlying multiyear ${\text{n}}\acute{{\text{e}}}{\text{v}}\acute{{\text{e}}}$/firn. 26 January 2018, Anchorage Island (67.6°S). Bloom shown was approximately 50 m × 100 m.

In the limited terrestrial ecosystems of Antarctica, all photosynthetic organisms will make a significant contribution to the ecology of their habitat. Ice-free ground makes up only around 0.18% of Antarctica’s continental area, and even in the Antarctic Peninsula, the most vegetated region of Antarctica, only 1.34% of this exposed ground is vegetated^1,2^. Photosynthetic life is not restricted to bare ground however, with algal blooms often appearing in coastal snowfields as green (Fig. 1) and red patches below and on the snow surface^3–5^. Blooms of snow algae in Antarctica were first described by expeditions in the 1950s and 1960s^6,7^ and have since been studied at a few locations in Antarctica, where they have been shown to host a diverse range of algal species^3,4,8–10^ and to play key roles in nutrient and carbon cycling^11–13^. Considering that a single snow algal bloom can cover hundreds of square metres^4^, snow algae are potentially one of the region’s most significant photosynthetic primary producers, as well as influencing nutrient provision to downstream terrestrial and marine ecosystems^14^.

Warming in the Antarctic Peninsula has already exceeded 1.5 °C over pre-industrial temperatures^15^, and current Intergovernmental Panel on Climate Change (IPCC) projections indicate further global increases^16,17^. Set against a background of natural decadal temperature variability^18,19^, climatic changes on the Peninsula are already influencing its vegetation^20,21^. With the available area for plant colonisation on the Peninsula likely to increase by up to threefold due to this warming^22^, understanding how snow algae fit into Antarctica’s biosphere and their probable response to warming is critical to understanding the overall impact of climate change on Antarctica’s vegetation.

Satellite remote sensing offers a step change in our ability to map and monitor the extent of Antarctica’s terrestrial biosphere. However, current remote sensing estimates of vegetation biomass and distribution are biased towards plants on exposed ground^1,23,24^ and often exclude snow algae from analysis as their spectral profile precludes the use of classical vegetation indices. Efforts to use remote sensing to identify and quantify snow algae have to date focused on the Northern Hemisphere, with early work using airborne hyperspectral imaging^25^ and newer predictive models developed for quantifying biomass and the bioalbedo (the impact of biological impurities on ice and snow albedo) of snow and ice^26–28^. Several studies have used satellite observations to investigate snow and ice algae on larger scales^29–31^, implicating algal blooms as significant drivers for darkening and enhancing melt of the Greenland ice sheet^31^. Current spectral and spatial resolution of freely available multispectral satellite imagery limits the study of most snow and ice algae to presence detection through classification models or assessing relatively small, ground validated areas. Large-scale observations are also hampered by strong forward scattering of light on snow, mountainous terrain and low solar zenith angles in the Polar regions, which introduce strong directional biases within satellite imagery, added to which frequent cloud cover and summer snowfall often obscure algae on the surface.

To mitigate these challenges, we make use of multiple years of data obtained from the European Space Agency’s (ESA) Sentinel 2 constellation of multispectral imaging satellites to provide the first estimate of the distribution, size and biomass of snow algal blooms across the entire the Antarctic Peninsula. To validate our approach, remote sensing was combined with in situ measurements of spectral reflectance factors, cell concentration, dry biomass, gas exchange and nutrient status, with data being collected over two field seasons, at Ryder Bay, Adelaide Island (67°S), in the 2017/18 summer, and the Fildes Peninsula, King George Island (62°S), in the 2018/19 summer. We show that the Antarctic Peninsula supports at least 1.3 × 10^3^ tonnes (dry mass) of green snow algae, covering approximately 1.9 km^2^. We also present data on the likely factors controlling snow algal distribution and discuss how this may be influenced by climatic warming.

## Results and discussion

### Ground validation campaign

**Fig. 2:**
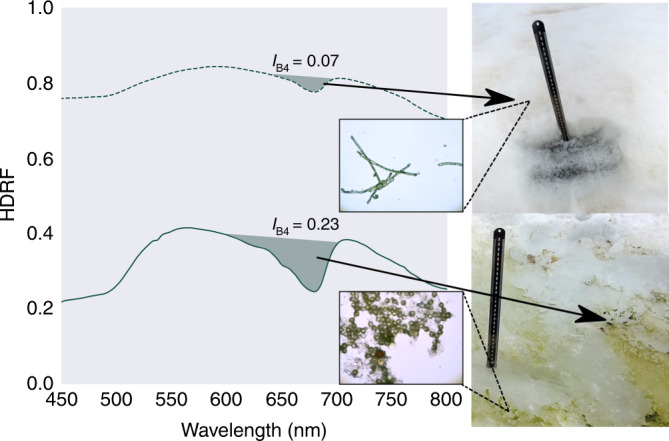
Snow algae reflectance factors. Hemispherical directional reflectance factors (HDRFs) of green snow algae, showing representative spectra from high (solid, green line) and low (dashed, green line) cell density patches of snow algae with corresponding *I*_B4_, sampling site photograph and brightfield microscope image (black scale bar represents 5 μm). HDRFs, photographs and microscopy from snow sampled close to Refugio Collins, King George Island, Antarctica.

Coastal snow fields at both field sites had visible blooms of green and red snow algae (see Fig. 1), ranging from 10s of cm^2^ to 100s of m^2^ in area. Early in the melt season (December/January), green snow algae was primarily observed within a band of slush between the seasonal and perennial snow layers. By February, large areas of this seasonal snow cover had melted and exposed the underlying green algae as a thin (c. 9 mm) layer on the surface of the underlying, older snow. Brightfield microscopy revealed the morphology of the green algae present, which ranged from unicellular round or elongate-ellipsoidal (single or clumped) to filamentous strands of cells (see Supplementary Fig. 1 for brightfield images). Hemispherical directional reflectance factors (HDRFs) were recorded for green snow algae on King George Island using a field spectrometer (Fig. 2). Significant variation in intensity of reflectance factors was observed across patches of green snow algae, with average visible/near infrared HDRFs for high cell density blooms c. 20% of control plots with no visible colouration. This lowering of albedo relates to increased absorption of light directly by algal cells as well as indirect influences, such as greater liquid water content in snow containing algae^26^. All HDRFs from green snow algal blooms (*n* = 91) featured characteristic chlorophyll *a* absorbance centred around 680 nm^25,26^ (see Fig. 2). This meant that they returned positive values of *I*_B4_ (Eq. (1)) when convolved to the spectral response of Sentinel 2’s multispectral imager. Values of *I*_B4_ derived from field-measured HDRFs ranged from 0.02 (measured cell density: 1.2 × 10^4^ cells ml^−1^) to 0.39 (measured cell density: 1.2 × 10^5^ cells ml^−1^).

Red and/or orange snow algae blooms (such as *Sanguina* sp., *Chloromonas polyptera* and *Hydrurus* sp.) are also a dominant ecosystem alongside, or even incorporated within, some green blooms^4,9,10,32^. However, in spite of their importance such dominant red or orange blooms had to be excluded from our study as absorbance from secondary carotenoids such as astaxanthin, present in red but not all green cells^33^, reduces the reflectance of Band 3 and flattens any chlorophyll absorbance feature within Sentinel 2 bands, making them difficult to detect automatically (see Supplementary Fig. 2). Although it is possible to relate secondary carotenoid absorbance to snow algae biomass^29^, broad absorbance below 500 nm is also indicative of mineral dust within the snow^25,26^, making it an unsuitable semi-automatic method to assess red snow algae. It is probable that green blooms detected within Sentinel 2 imagery also contain red and/or orange cells, but due to the resolution we can only base the findings on the chlorophyll pigments and so assume that these blooms are green dominant.

**Fig. 3:**
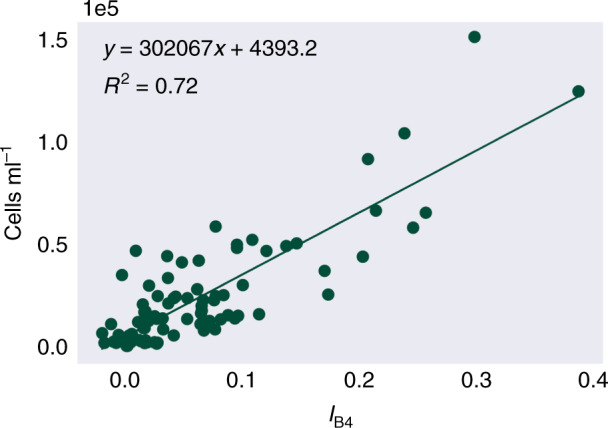
Cell density versus *I*_B4_. Linear regression of the scaled integral of Sentinel 2's Band 4 relative to Bands 3 and 5, versus concentrations of green algal cells within the snow (*n* = 91).

The linear relationship between cell density and I_B4_ (Fig. 3) was high and significant, with a Pearson’s correlation coefficient of *r*(89) = 0.85, *P* < 0.01. Likely causes of variation include factors that affect the HDRF lineshape, such as debris and/or red algal cells within the snow, and snow morphology, such as crystal structure and liquid water content^27^. Variation may also be derived from sampling geometry, with a fixed nadir viewing angle, but with aspect, slope angle and the solar zenith varying between sampling sites. The *y*-intercept of the linear regression model from Fig. 3 determined the lower limit of detection of green snow algae within a Sentinel 2 pixel, 4.4 × 10^3^ cells ml^−1^. However, as seen in Fig. 1, blooms were typically not homogeneous at the 10 × 10 m scale of a Sentinel 2 pixel. On snow, any chlorophyll absorbance from algae will be integrated across a pixel according to its point spread function^34^ with a theoretical minimum area limit of detection based on the bloom’s cell density and whether it crosses through the centre or is positioned at the border of a pixel. Combining and averaging green snow algae and white surface snow HDRFs from our 10 × 10 m sampling grid and assuming a bloom crosses through the centre of a pixel, we empirically estimate the minimum Sentinel 2-detectable bloom area to be 11 m^2^. Mixed pixels containing rock or vegetation alongside green snow algae would likely be excluded from the study based on the filter functions in Eq. (3).

### Green snow algal biomass

**Fig. 4:**
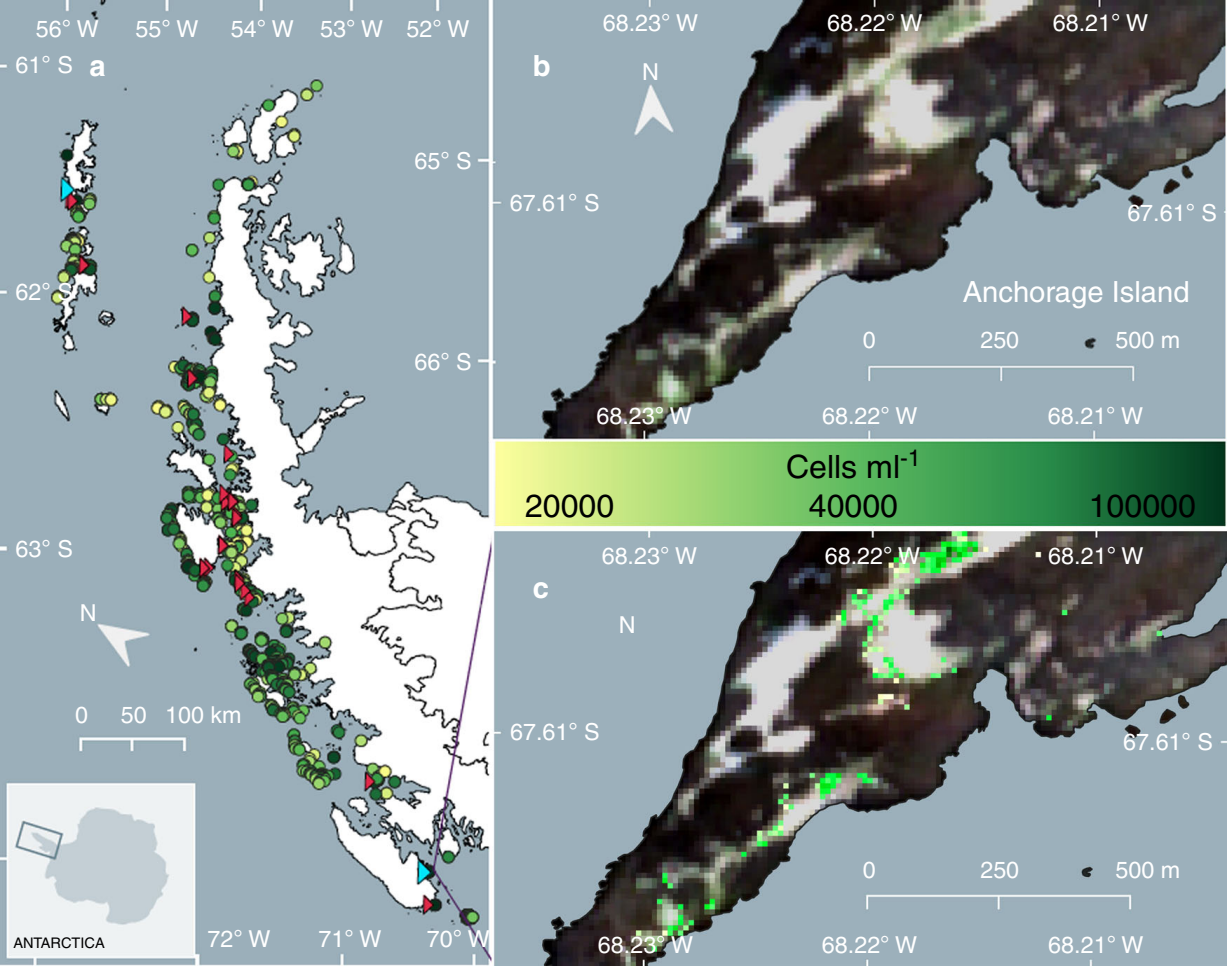
Green snow algae distribution and modelled cell density. **a** Overview of the locations of individual blooms of green-dominant snow algae identified across the Antarctic Peninsula using modelled data from satellite imagery and ground data (circles; *n* = 1679). Circle colour scale represents the mean cell density (cells ml^−1^) of each bloom. Red triangles indicate the location of ground validation sites (*n* = 27). Cyan triangles show the location of our Adelaide Island and King George Island field sites. **b** RGB Sentinel 2A image of green snow algae blooms at one of our validation sites, Anchorage Island (February, 2020). **c** Output of *I*_B4_ (Eq. (1)), where coloured pixels are those not masked by Eq. (3). Pixel values are converted to cell density (cells ml^−1^) using Eq. (2) with the colour scale showing the resultant cell density for each pixel identified as containing green snow algae.

Summer Sentinel 2A and 2B imagery of the Antarctic Peninsula from 2017, 2018 and 2019 was used to produce the first estimate of snow algal biomass distribution for the region. Figure 4 shows the first Antarctic Peninsula scale map of the distribution and average cell concentration estimates of green snow algae. Remote-sensed locations were validated using bloom sightings from published literature^7,9,14,35^, a visitor survey at the 2018 Scientific Committee of Antarctic Research (SCAR) Open Science Conference^36^, images collected by Antarctic researchers and those available on the Secretariat of the Antarctic Treaty’s visitor site guidelines website^37^. Validation sites are shown as red triangles in Fig. 4 and our remote-sensed bloom locations have a kappa score of 0.81 (*n* = 25) when referenced against these observations.

In total, 1679 individual blooms of green snow algae were identified. A large range in the area of individual blooms was observed, averaging 1043 m^2^, but spanning 300 m^2^ (based on our lower area filter) to 145,000 m^2^, the latter observed on Robert Island, South Shetland Islands (62.4°S). In total, 1.9 × 10^4^ pixels were identified as containing green snow algae, covering 1.9 km^2^ of the total area of the Antarctic Peninsula studied here (c. 2.7 × 10^5^ km^2^). For comparison, commensurate, high confidence (Landsat imagery; NDVI > 0.1) estimates of the area covered by other terrestrial vegetation on the Peninsula, is 8.5 km^2 ^^1^. Pixel cell concentrations also varied significantly, ranging between 1.9 × 10^4^ cells ml^−1^ and 1.7 × 10^5^ cells ml^−1^. These results compare well with our in situ measurements (averaging 2.2 × 10^4^ cells ml^−1^; see Table 1) but are lower than values reported elsewhere (e.g. 1.2 × 10^6^ cells ml^−1^)^4^, likely because our sampling strategy aimed to capture variation on a 10 × 10 m Sentinel 2 pixel scale. A number of factors cause uncertainty in the presented area estimates as, although our method will detect subpixel blooms, it will integrate the actual cell density across the area of the entire pixel, hence overestimating the visible area of algae in this scenario. Conversely, our output is derived from a snap shot of seasonal growth, and other green algae will be obscured from view by overlying snow. Widespread field surveys combined with high resolution, frequent revisit satellite imagery would be necessary to address these limitations and should be the focus of future investigations.

We combined remote-sensed observations with in situ measurements to estimate snow algal biomass (dry mass). Based on the bloom area, the average thickness of green algal blooms on the snow surface and the density of the snow algal surface layer (Table 1), we normalised per pixel cell concentrations by area (see Table 1 for average cells m^−2^ values). Green snow algae biomass was then calculated using these cell-area concentrations along with the average in situ measured dry mass of a green snow algae cell (2.4 × 10^−8^ ± 2.2 × 10^−8^ g). Propagated error from this calculation results in an uncertainty of plus 564% and minus 5% relative to biomass values reported here using mean values. Algal biomass estimates from Sentinel 2 imagery ranged from 5 to 5791 g dry mass m^−2^ (averaging 58 g dry mass m^−2^), comparing well with in situ measurements (averaging 30 g dry mass m^−2^; see Table 1) and spanning a range similar to that caused by propagated error. Combining average biomass estimates from all identified blooms, green snow algae had a total annual dry biomass of 1.3 × 10^3^ tonnes on the Antarctic Peninsula, which, based on average %C content of green snow algae (Table 1), is equivalent to 479 tonnes of carbon within a growth season. Future work should prioritise incorporating red snow algal blooms into this figure, as, though field measurements suggests that red blooms contain less mass on a per m^−2^ basis (averaging 12 g dry mass m^−2^ ± 19), field observations indicate red snow algae are likely to cover at least half the area of green snow algae (average measured area of Ryder Bay red algal blooms = 328 m^2^ versus 714 m^2^ for green blooms) and would significantly increase total biomass estimates. In addition, the carbon content of previous green and red blooms at the start of the season is also largely unknown.

**Table 1:** Snow algae biogeochemistry.

	Ryder Bay (67°S)	King George Island (62°S)	Sentinel 2 remote-sensed data
Snow algae cells ml^−1^ snow melt	2.2 × 10^4^ ± 2.4 × 10^4^ (*n* = 56)	2.2 × 10^4^ ± 3.1 × 10^4^ (*n* = 35)	4.2 × 10^4^ ± 1.3 × 10^4^ (*n* = 17,520)
Snow algae cells m^−2^ snow surface	3.9 × 10^9^ ± 8.8 × 10^9^ (*n* = 56)	2.2 × 10^9^ ± 2.7 × 10^9^ (*n* = 35)	2.2 × 10^9^ ± 6.9 × 10^9^ (*n* = 17,520)
Snow algae community dry mass g m^−2^	51.3 ± 44.0 (*n* = 19)	16.2 ± 21.2 (*n* = 31)	57.9 ± 173.0 (*n* = 17,520)
Green snow density (ml melt cc^−1^ snow)	0.56 ± 0.17 (*n* = 35)	0.59 ± 0.16 (*n* = 55)	0.58^a^
Snow algae layer thickness (mm)	12.7 ± 6.3 (*n* = 35)	7.2 ± 5.6 (*n* = 55)	9.05^a^
NCER (μmols CO_2_ m^−2^ s^−1^)^b^	−0.099 ± 0.099 (*n* = 3242)	−0.037 ± 0.029 (*n* = 1158)	−0.064 ± 0.19^c^
ER (μmols CO_2_ m^−2^ s^−1^)	0.089 ± 0.125 (*n* = 846)	−0.016 ± 0.02 (*n* = 195)	—
GEP (μmols CO_2_ m^−2^ s^−1^)	−0.188	−0.021	—
Snow algae %C	41.8 ± 8.8 (*n* = 57)	23.1 ± 10.2 (*n* = 25)	36.1^a^
Snow algae %N	6.2 ± 1.6 (*n* = 64)	3.3 ± 2.2 (*n* = 25)	—
Snow algae *δ*^15^N (‰)	11.4 ± 6.5 (*n* = 61)	−2.1 ± 2.4 (*n* = 25)	—
Green snow nitrate (μmols l^−1^)	15.64 ± 12.26 (*n* = 42)	—	—
Green snow phosphate (μmols l^−1^)	11.06 ± 13.69 (*n* = 19)	—	—

In situ cell counts, biogeochemistry and carbon flux of Antarctic green snow algae compared to remote-sensed estimates. Reported values are mean ± 1 standard deviation and are from field work conducted in the Ryder bay area of Adelaide Island (2018) and the Fildes Peninsula area of King George Island (2019).

*NCER* net carbon exchange rate, *ER* ecosystem respiration, *GEP* gross ecosystem photosynthesis.

^a^Average used in biomass model.

^b^Negative values denote carbon flux from the air into the snow ecosystem and positive values for flux from the snow to the air.

^c^Based on modelled biomass produced per m^2^.

Since the snow algae blooms identified within satellite imagery are the surviving product of a summer’s growth, we can use our biomass estimates to infer rates of seasonal carbon uptake. Assuming a 122-day season and a 17-h period of photosynthesis (based on average growth hours recorded for in situ carbon flux chamber measurements), snow algae would need an average net carbon exchange rate (NCER) of −0.064 μmols CO_2_ m^−2^ s^−1^ to build up the observed biomass, similar to measured in situ rates (average of −0.08 μmols CO_2_ m^−2^ s^−1^; see Table 1). Rates of ecosystem respiration (ER) from Ryder Bay and King George Island (average of 0.07 μmols CO_2_ m^−2^ s^−1^; see Table 1) indicated that snowpack heterotrophs, bacteria and fungi^4,38,39^ were also active and producing CO_2_ within the snowpack. NCER at snow algal blooms, however, was negative across a range of sunlight conditions (photosynthetically active radiation (PAR) ranging from 9 to 2594 μmols m^−2^ s^−1^, averaging 398 μmols m^−2^ s^−1^), and we therefore suggest green snow algae to have positive net ecosystem production (i.e. a short-term net sink of carbon until biological degradation occurs or the algae are eaten) throughout the summer season. Compared to other terrestrial vegetation, calculated in situ rates of gross ecosystem photosynthesis (GEP) (Table 1) were similar to other plant species in Antarctica^39,40^. However, flux measurements from other Antarctic plant ecosystems indicate complexity in net carbon exchange. High rates of soil-based microbial respiration (0.27–2.23 μmols CO_2_ m^−2^ s^−1^)^39,40^ can lead to vegetated sites being net sources of CO_2_ (−0.03 to 0.62 μmols CO_2_ m^−2^ s^−1^)^39^, though well-established vegetation shows largely negative NCER over a growth season^40^. This highlights a need for large-scale characterisation of carbon fluxes from Antarctic terrestrial vegetation, including snow algae, and their associated heterotrophic communities and is especially pressing considering observed increases in growth rates in response to Antarctic warming^41–43^.

### Snow algae distribution controls

Understanding controls on the distribution of snow algae is crucial for predicting how blooms may respond to the future warming of the coastal zone of Antarctica, forecast by models in the Fifth Climate Model Intercomparison Project^44^. Snow algae require liquid water, light and nutrients to grow, yet our understanding of how they respond to variability in these different factors is limited to in vitro experiments^45,46^ or analysis of snow algae metabolites^4,47^. Mapping snow algal biomass at large scale along the Antarctic Peninsula provides an opportunity to explore some of these controls based on geospatial relationships. The blooms identified in Fig. 4 were predominantly in coastal snowfields on the western side of the Peninsula and occurred over a latitudinal range of 62.3°S–68.1°S. The South Shetland Islands (62.3°S) were the northern-most outlying islands considered in this study, though blooms certainly occur further north on the South Orkney and South Sandwich Islands, and on Sub-Antarctic South Georgia^48^. Our most southerly observation was on the Faure Islands at (68.1°S). Data from the SCAR-READER near-surface air temperature database^19^ and the 2-m Regional Atmospheric Climate Model (RACMO2.3)^49^ indicate that this latitudinal range (62°S–68°S) corresponds with a region of the Peninsula that experiences average summer air temperatures >0°C, implying seasonal snow melt and the availability of liquid water within this zone. We see similar temperature zonation when relating snow algae-containing pixels to elevation using the 8-m Reference Elevation Model for Antarctica (REMA)^50^, with the majority of blooms occupying low lying snowfields (averaging 14.8 ± 9.0 m above sea level) and infrequently occurring at higher elevations. The majority of blooms were on flat or moderately sloping snow surfaces, with the average slope being 14.5° ± 12.9°. Only smaller blooms were observed on steeper ground (up to 72.8°), with blooms >1300 m^2^ absent on slopes >30°, indicating that snow instability and/or enhanced wash out of snowpack nutrients^51^ may prevent large blooms forming on steeper slopes. No trends were observed for aspect, with blooms occupying snow facing all directions. This may be expected given the typically cloudy, diffuse light and long-day conditions over the Peninsula.

Marine fauna are a potential source of nutrients for Antarctic snow algae, with faeces at seal haul-outs, penguin colonies and nesting sites for other birds providing hot spots of nitrogen and phosphate in an otherwise typically oligotrophic environment^14,35,51–54^. Indeed, our Ryder Bay green snow algae sites were in proximity to elephant seal wallows and skua and kelp gull nesting sites. Our sites contained elevated nitrate and phosphate concentrations relative to inland values recorded by Nowak et al.^54^ for the same locality, as well as enriched δ^15^N, indicative of nitrogen inputs from higher trophic levels^35,52,53^ (Table 1). The influence of marine fertilisation was also evident in our Peninsula-wide survey, with 49% of observed blooms being within 100 m of the sea, and 60% of blooms being within 5 km of a penguin colony^55^. Moreover, the average area was larger (1257 m^2^ versus 960 m^2^; *t* test: *t* = 1.4; *P* < 0.16) and mean cell concentration significantly larger (4.1 × 10^4^ cells ml^−1^ versus 3.7 × 10^4^ cells ml^−1^; *t* test: *t* = 6.4; *P* < 0.01) at the 30% of blooms <1 km from a penguin colony relative to those outside this radius, suggesting that nutrients supplied by Antarctic marine fauna are utilised by snow algae and influence growth rates. This is a significant finding because measured %N of green snow algae collected from Ryder Bay and King George Island (Table 1) implies an annual nitrogen requirement of 71.7 tonnes to support the observed Peninsula-wide growth of green snow algae; roughly equivalent to 3.1 g of bioavailable nitrogen being supplied per m^2^ of snowpack in a growth season. Based on the nitrogen content measured at our Ryder Bay sites (Table 1) and values reported elsewhere^14,51,54^, this would necessitate a resupply of nutrients throughout the melt season, either through melt-out and mobilisation of nutrients within a larger area of snow or added windblown/direct inputs from sources such as marine fauna.

### Implications for a warming Antarctic Peninsula

Our study indicates that positive summer temperatures and a sufficient nutrient supply are key factors determining the present-day distribution of green snow algae on the Antarctic Peninsula. With the IPCC’s projected 1.5 °C global temperature increase, it is predicted that the 0 °C isotherm will increase in elevation and that positive degree days will become more commonplace and occur further to the south^56^. This will likely open up new snow for colonisation by green snow algae, should an appropriate dispersal mechanism allow transfer to new areas. The impact warming would have on marine nutrient supply to the snowpack is less clear, as marine vertebrates have shown varying degrees of plasticity in response to a changing Antarctic environment^57,58^. Southern expansion of marine habitats could increase the number of nutrient hotspots in the south, yet stresses resulting from increased precipitation or food chain disruption may negatively impact established bird populations^58,59^.

**Fig. 5:**
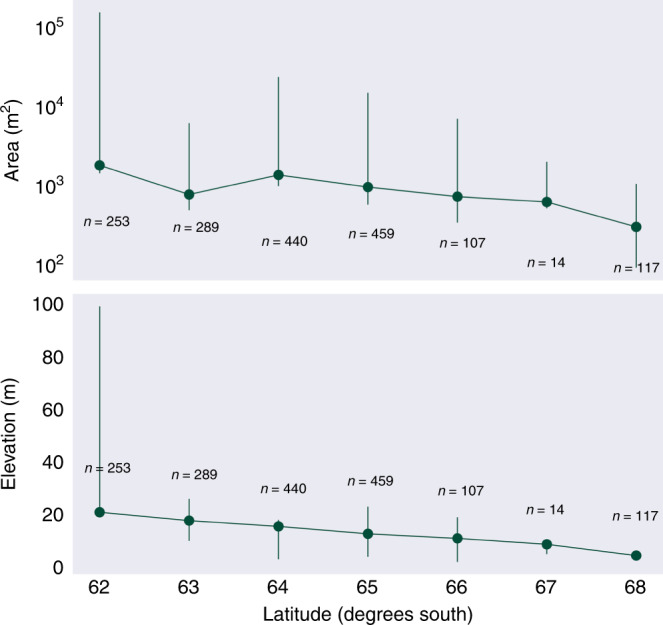
Spatial trends of green snow algae. Mean green bloom area averaged per degree latitude with logarithmic area scale. Mean bloom elevation above sea level averaged per degree latitude. Error bars report range (minimum and maximum values).

The latitudinal range over which we currently observe green snow algae provides a small summer temperature gradient (1.5 °C at Bellinghausen Station (62.2°S) to 0.5 °C at San Martin Station (68.1°S)^19,49^; J/F/M average) and we observe both average area and maximum bloom elevation increasing towards the north of Peninsula (Fig. 5). High maximum area and elevation observations for 62°S relative to the rest of the Peninsula (Fig. 5) was the result of two very large blooms in the South Shetland Islands, at Robert Island (62.4°S) and Nelson Island (62.3°S) (shown in Fig. 4). Both blooms occurred adjacent to and downwind of large chinstrap and gentoo penguin colonies^55^, and both islands have large ablation zones extending high up onto their local ice caps. Robert Island had both the largest observed bloom area and supported the highest elevation observation of green snow algae (99 m above sea level), whereas the Nelson Island bloom contained the highest observation of biomass (2.1 kg C m^−2^). Among the most northern of our observations, these blooms could be used as a model for change as the Peninsula warms and that, at least in the short term, an increase in ablation zone area may facilitate bloom area increases at sites with large bird or seal populations to supply this new habitable snow with nutrients. However, 62% of blooms observed in this study were on small islands with no local ice caps or mountains to allow upward range expansion, and a warming Peninsula could see a loss of summer snow on these islands (unless they are able to bloom earlier in the season). In our snapshot of blooms on the Peninsula, 95% of the observed green snow algal biomass comes from relatively few (0.05%) large blooms, and the contribution from these low-lying islands was small, comprising only 0.004% of total observed biomass. A warming Peninsula, therefore, may see a shift towards fewer, larger snow algae blooms, resulting in a significant increase in biomass on larger outlying islands and the mainland. The coupled loss of blooms from smaller islands would be insignificant with respect to biomass and may be mitigated by southward range expansion or an earlier growth season. However, with multiple and often unknown species recorded within patches of green snow algae^4,8–10,52^, and little known about the dispersal mechanisms, life cycles and plasticity of snow algal species, losses from these islands could represent a reduction of terrestrial diversity for the Antarctic Peninsula.

## Methods

### Remote sensing model development and validation

We used a scaled integral approach adapted from Painter et al.^25^ to quantify snow algae within Sentinel 2 imagery. This relates the spectral reflectance profile within pixels to chlorophyll absorption and is less sensitive to bidirectional reflectance distribution function (BRDF) effects between images as it is based on the area of a chlorophyll absorption feature rather than its depth^60,61^. BRDF effects are the result of strong forward scattering of light on snow, complex, mountainous terrain and low solar zenith angles in Antarctica and caused large uncertainties when using spectral unmixing and physical classification methods to identify snow algae across the 3-year data set required to image the entire Antarctic Peninsula.

**Fig. 6:**
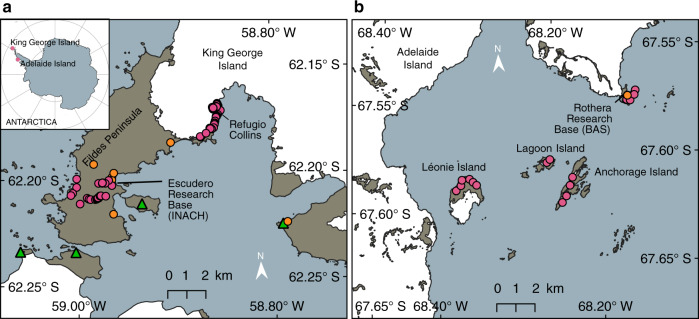
Ground validation sites. Pink circles show sampling locations from which snow algae hyperspectral hemispherical directional reflectance factors (HDRFs) were recorded and algae/snow taken for biogeochemical analysis. Orange circles denote the location of research bases and green triangles denote the location of penguin colonies^55^. **a** Sampling conducted in the Fildes Peninsula region of King George Island while stationed at Profesor Julio Escudero Base and Refugio Collins in 2019. **b** Sampling conducted in the Ryder Bay area of Adelaide Island, showing Rothera Research Base and the outlying islands from which snow algae were sampled in 2018. Map data from the SCAR Antarctic Digital Database.

Painter’s approach estimates algal biomass by scaling the integral of chlorophyll absorbance by its continuum^25^, but is based on red snow algae and hyperspectral imagery. We used field spectroscopy to develop our own regression model and relate chlorophyll absorbance within Sentinel 2 bands to the concentration of green snow algae cells observed within Antarctic snow fields from the Ryder Bay/Rothera area (68°S) in 2018 (95 samples) (see Fig. 6b) and the Fildes Peninsula area of King George Island (62°S) in 2019 (91 samples) (see Fig. 6a). We adopted a grid sampling strategy to capture spatial variation at a 10 × 10 m scale to replicate the ground sampling distance of Sentinel 2. Where identified, blooms (16 individual blooms in total) were subdivided and 10 × 1 m lateral and 10 × 1 m longitudinal patches, their GPS position was logged using a Trimble 5700 GPS receiver and Zepher Antenna (Ryder Bay) and an Emlid RS+ GNSS receiver (King George Island). Visible bloom area was measured using a tape measure, and the temperature, time of day, PAR and aspect of slope were recorded at the time of sampling. For Ryder Bay sampling, an Spectra Vista Corporation (SVC) 1024i field spectrometer with 14° field of view (FOV) foreoptic was used to collect 3×  hyperspectral HDRFs^62^ from each patch. HDRFs were recorded under clear sky conditions at a nadir viewing angle with a 98% Spectralon panel used as a white reference between each HDRF measurement. The sampling protocol outlined in Cook et al.^27^ was adopted for both field campaigns. Fixed viewing geometry ensured that HDRF was recorded consistently over a 908-cm^2^ FOV. The snow in the FOV was subsequently sampled into a sterile 50-ml falcon tube, with care taken not to compress the snow into the tube. The samples were then transferred to the Bonner Laboratory (Rothera Research Station, Ryder Bay, Antarctica) or to the Profesor Julio Escudero Base laboratory (King George Island (KGI), Antarctica) for processing. Field samples were collected at Ryder Bay under the UK BAS Operating Permit and the Antarctic Act (1994; 2013) and at KGI under permit from INACH (Chile) Certificate number 209/2019.

Within the research station laboratory, samples were melted in 4 °C lit incubators (Sanyo), with their melted volume recorded and used to calculate snow density. Algal community dry cell mass was obtained by gravity filtration of 50 ml of melted snow through a pre-weighed filter (Whatman GF/C, 47 mm). Filters were dried at 80 °C for at least 48 h prior to re-weighing. Determination of nitrate and phosphate (as orthophosphate, PO${}_{4}^{3-}$) concentration was performed colorimetrically using a Hach Lange DR 3900 spectrophotometer with the appropriate test kits (Nitrate Kit LCK 339, range 1–60 mg l^−1^; Phosphate Kit LCK 349, range 0.15–4.5 mg l^−1^, Hach Lange, Manchester, UK)^63^. Samples for total carbon and nitrogen were processed by pelleting 2 ml of snowmelt (2000 g for 10 min at 4 °C), discarding the supernatant and drying the pellet at 80 °C for 24 h before transfer to the UK by ship for further analysis. These were analysed for %carbon, %nitrogen and ^14^N/^15^N using a Costech Elemental Analyser attached to a Thermo DELTA V mass spectrometer in continuous flow mode. Precision of analyses is  ±0.5% for C and N and better than 0.1‰ for ^14^N/^15^N. The above values are presented in Table 1.

Cell density was determined through analysis of colour brightfield microscope images in ImageJ2^64,65^. To obtain the brightfield images, melted snow samples that were preserved in 2% formaldehyde at the Bonner Laboratory were mixed by gentle shaking followed by pipetting a 15-μl sub-sample directly onto the haemocytometer (Neubauer-improved) and imaged using a Leica DM600B microscope. To count the cells, a 5 × 4 grid square was drawn using the haemocytometer grid lines on the brightfield image and cropped (Supplementary Fig. 3a). Images were then converted to 8-bit greyscale and a threshold was applied (default, B&W) so that the cells appear black on a white background. The ‘despeckle’ function was used to remove background noise (Supplementary Fig. 3b). The ‘set scale’ function was used by tracing the scale bar on the image to additionally determine cell size in μm^2^. Cells (including any residual extracellular polymeric substances and mineral debris) were automatically counted using the ‘analyse particles’ function using a size range of 0–infinity μm^2^ and circularity of 0.00–1.00 (Supplementary Fig. 3c). See Supplementary Fig. 3 for ImageJ output. On average, 6% (14% SD) of the cells in the 60 green-dominant samples that were collected during the ground validation at Ryder Bay were visually considered to be red or orange.

Hyperspectral HDRFs were convolved to the spectral response of Sentinel 2A and absorption from chlorophyll measured as the scaled area-integral of Band 4 (665 nm) relative to Bands 3 (560 nm) and 5 (705 nm) using Eq. (1) (see Fig. 2 and Supplementary Fig. 2 for visual representations of *I*_B4_.

1 $${I}_{{\rm{B}}4}=\int_{{\lambda }_{{\rm{B}}3}}^{{\lambda }_{{\rm{B}}5}}\frac{{R}_{{\rm{Cont}}_{{\lambda }_{{\rm{B}}4}}}-{R}_{{\rm{Snow}}_{{\rm{B}}4}}}{{R}_{{\rm{Cont}}_{{\lambda }_{{\rm{B}}4}}}}{\rm{d}}\lambda$$

where *I*_B4_ is the integral of Band 4, ${R}_{{\rm{Cont}}_{{\Lambda }_{{\rm{B}}4}}}$ is the HDRF of the continuum between Bands 3 and 5, interpolated to the centre wavelength of Band 4, ${R}_{{\rm{Snow}}_{{\rm{B}}4}}$ is the measured HDRF of Band 4, and ${\lambda }_{{{\rm{B}}}_{n}}$ is the wavelength at the centre point of Band ‘*n*’. As in Painter et al.^25^, the linear regression (Fig. 3) of *I*_B4_ versus measured algal cell density within the field spectrometer’s FOV was used to relate *I*_B4_ within a Sentinel 2 pixel to algal cell concentration in snow within imagery of the Antarctic Peninsula. The expression to estimate the cells ml^−1^ of snow melt within a Sentinel 2 pixel was derived from the line of best fit (Eq. (2) (n = 91; *R*^2^ = 0.72)).

2 $${\mathrm{Cells}}\ {\mathrm{ml}}^{-1}=({I}_{{\mathrm{B}}4}{\times}302067)\,{+}\,4393$$

### Sentinel 2 imagery analysis

Green snow algal biomass across the Antarctic Peninsula was estimated by applying Eqs. (1) and (2) to Sentinel 2A and Sentinel 2B imagery. Coverage from King George Island (62°S) to Eklund Islands (72°S) was achieved by combining 2017, 2018 and 2019 February/March imagery with <20% cloud cover. There was a notable gap in the data, with no suitable cloud-free imagery covering the north of King George Island, west of Livingston Island, Deception, Snow and Smith Islands. Atmospheric correction, cloud masking and BDRF correction was performed on Level 1C imagery using Sen2Cor processor (ESA’s Sentinel Application Platform (SNAP; 6.0.0)). During fieldwork in Ryder Bay and King George Island, it was observed that green snow algae would typically occur within wet snow at the boundary between the new snow layer and older underlying névé or firn, and melt out of the new snow would deposit green algae in a thin layer on the surface of the underlying snow, hence becoming visible to satellites (Fig. 1). By using imagery from February or March, we aimed to capture the Peninsula’s coastal snow fields in this condition, as algae exposed on the snow surface are easier to detect in remote sensing imagery, and though it is possible to detect chlorophyll absorption through overlying snow, we would be unable to estimate its biomass.

Applying Eq. (1) to Sentinel 2 imagery produced false positives, notably from other terrestrial vegetation, crevassed areas and mixed pixels. These were masked from analysis using the filter functions described in Eq. (3) that, tested against convolved field spectrometer data, would not filter out pure green snow algae pixels.

3 $$(B2 \, \ge \, B5) \, OR \, (B2 \, > \, B3) \, OR \, (B2 \, > \, 1) \, OR \, (B11 \, > \, 0.15) \, OR \, (B2 \, < \, 0.3 \, AND \, B8 \, < \, 0.25) \, \\ OR \, (B8 \, < \, B8a) \, OR \, (B4 \, > \, B5)$$

To reduce noise further, blooms were also filtered based on size and average biomass. Areas with fewer than three adjacent positive *I*_B4_ value pixels were excluded from analysis, as were pixels with an estimated biomass <4390 cells ml^−1^, the *y*-intercept of Eq. (2). This will have excluded some smaller patches of snow algae from analysis but was necessary to reduce the influence of false positives within our interpretation. Each pixel’s cells ml^−1^ estimate was converted to cells m^−2^ using average field observations of layer thickness (9.05 mm; *n* = 90) and snow density (0.58 ml melt cc^−1^ snow); *n* = 90) of the melt-accumulated algae/snow layer on the surface of old snow to estimate the liquid volume of snow in the known area of one pixel. Snow algal dry biomass was estimated using the average measured mass of a green algae cell (2.4 × 10^−8^ ± 2.2 × 10^−8^ g), determined by dividing cell density by blank-corrected dry mass for each sample. Blank correction used the average volumetric dry mass (9.6 × 10^−5^ ± 3.7 × 10^−5^ g; *n* = 9) of snow adjacent to green snow algal blooms but containing no visible algal cells under the microscope, as an estimate of the mass of non-algal components within the snow. The average percentage content of carbon and nitrogen (derived from C and N analysis) was also used to estimate the algal-based elemental mass of each.

Geospatial analysis was conducted using QGIS 3.6.2-Noosa and ArcMap 10.5.1, with comparative data sets being the REMA DEM^50^, RACMO 2m Annual Temperature Model^49^ and the Mapping Application for Penguin Populations and Projected Dynamics^55^ penguin colony database.

### Net carbon exchange rate

NCER was measured using an ADC Scientific Ltd (Herts, UK) LCPro-SD infrared gas analyser using a modified ADC Scientific Ltd clear plastic soil chamber. A clear chamber extension ring was placed into the snow through to the névé layer and sealed to the chamber. The CO_2_ was measured at a flow rate of 100 ml min^−1^, using ambient atmospheric CO_2_ collected from a distance of 3 m from the chamber, and under ambient natural light. PAR, temperature and NCER were collected at 1-min intervals. Chambers were placed over snow algae patches over nine separate days (totalling 51 h of measurements collected in the light and 16 h of measurements collected in the dark) from 22 January 2018 to 12 February 2018 at Ryder Bay and 2 days (totalling 16 h of measurements collected in the light and 6 h collected in the dark from 4 February 2019 to 9 February 2019 at King George Island. Data were collected at 1-min intervals, with respective conditions for Ryder Bay and King George Island sampling being: atmospheric CO_2_ concentration (μmols CO_2_ m^−2^ s^−1^): 403 (±5.5), 408 (±1.8); PAR (μmols m^−2^ s^−1^): 398 (±395, dawn to dusk variation), 488 (±202); chamber temperature: 6.1 °C (±4.8), 5.3 °C (±1.3). Measurements acquired in the light (>10 PAR) provide NCER values of photosynthesis minus ER (hence negative values as carbon is taken up by the community). Dark measurements (night or using blackout covers over the bloom) collected rates of ER only. Measurements from control sites, where no algae were visible in the snow, were used to record carbon fluxes from abiotic and heterotrophic activity. GEP was estimated by subtracting ER from NCER. All measurements were derived from the Ryder Bay and KGI bloom areas.

## Supplementary information

Supplementary Information

## Data Availability

Data that support the findings of this study are available to download at 10.6084/m9.figshare.c.4893771.
