# Supplementary material for: Remote sensing reveals Antarctic green snow algae as important terrestrial carbon sink
Source: Nat Commun. 2020 May 20;11:2527. doi: 10.1038/s41467-020-16018-w (PMC7239900; doi:10.1038/s41467-020-16018-w)
Supplement: Supplementary file 1 — Supplementary Information [file 41467_2020_16018_MOESM1_ESM.pdf]

Supplementary Information for "Remote sensing reveals Antarctic green snow algae as important terrestrial carbon sink"

Gray et al.

March 31, 2020

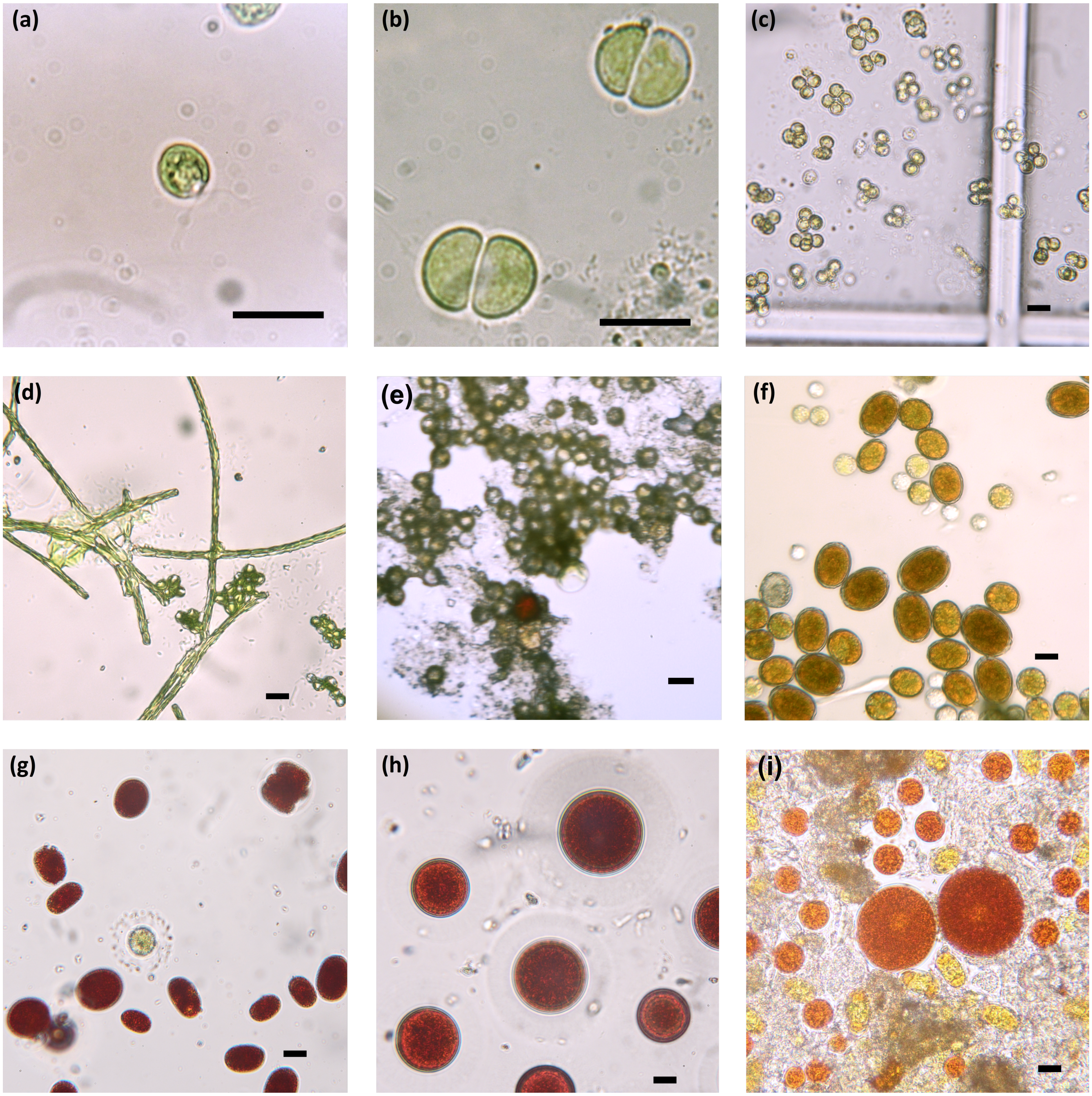

Supplementary Figure 1. Antarctic snow algal cells. Representative morphologies of green, orange and red algae from the community composition ecological survey from Ryder Bay and King George Island, Antarctica (cyanobacteria may be present in some samples). Cells ranged from unicellular round or elongate-ellipsoidal. Images viewed using Leica Laborlux 5 microscope at $\times 40$ or $\times 100$ magnification. Black scale bar represents 5µm. Samples from (a) Rothera Point, 2nd January 2018 (b) Rothera Point, 2nd January 2018 (c) Rothera Point, 19th January 2018 (d) Rothera Point, 14th January 2018 (e) Fildes Peninsula, King George Island, 10th February 2019 (f) Rothera Point, 14th January 2018 (g) Rothera Point, 29th January 2018 (h) Lagoon Island 5th February 2018 (i) Léonie Island 5th January 2018.

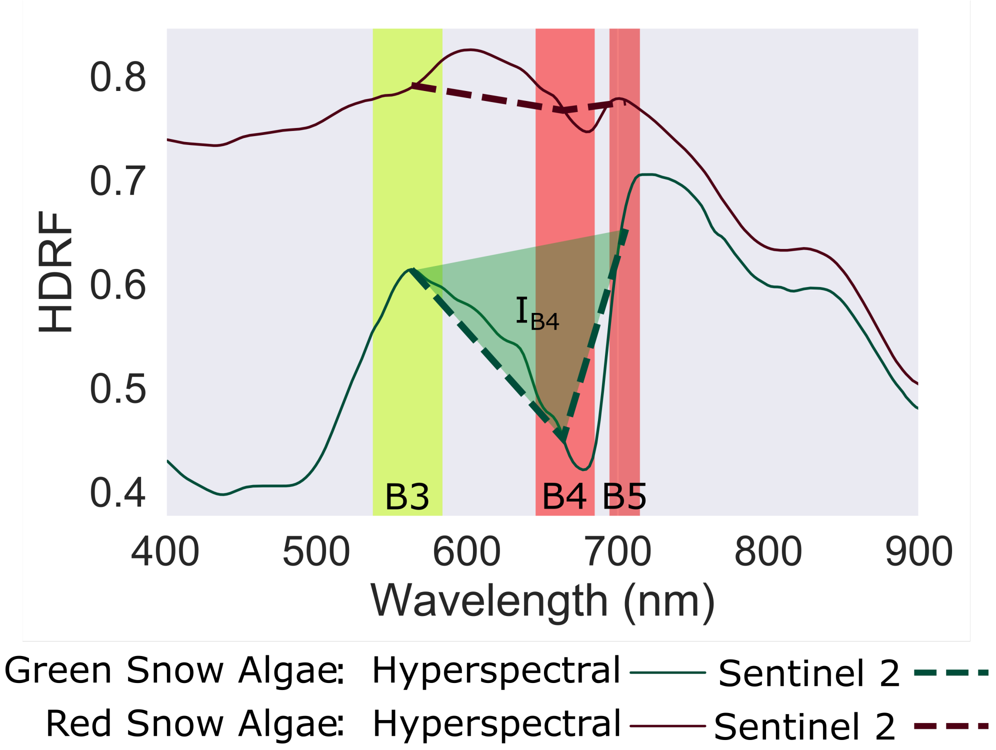

Supplementary Figure 2. Reflectance factors of snow algae. Average hemispherical directional reflectance factors (HDRF) of red snow algae (n=68) and green snow algae (n=91), from Ryder Bay and King George Island. Vertical bands indicate the spectral bandwith of Bands 3, 4 and 5 on Sentinel 2's multispectral imager. Bold dashed lines show the HDRF of red and green snow algae convolved to the spectral response of Bands 3, 4 and 5 of Sentinel 2 multispectral imager. The shaded area (I_{B4}) is described by the scaled integral of Equation 1, and relates to chlorophyll absorbance. Note how (I_{B4}) is affected by absorbance of secondary pigments within Band 3 for red snow algae.

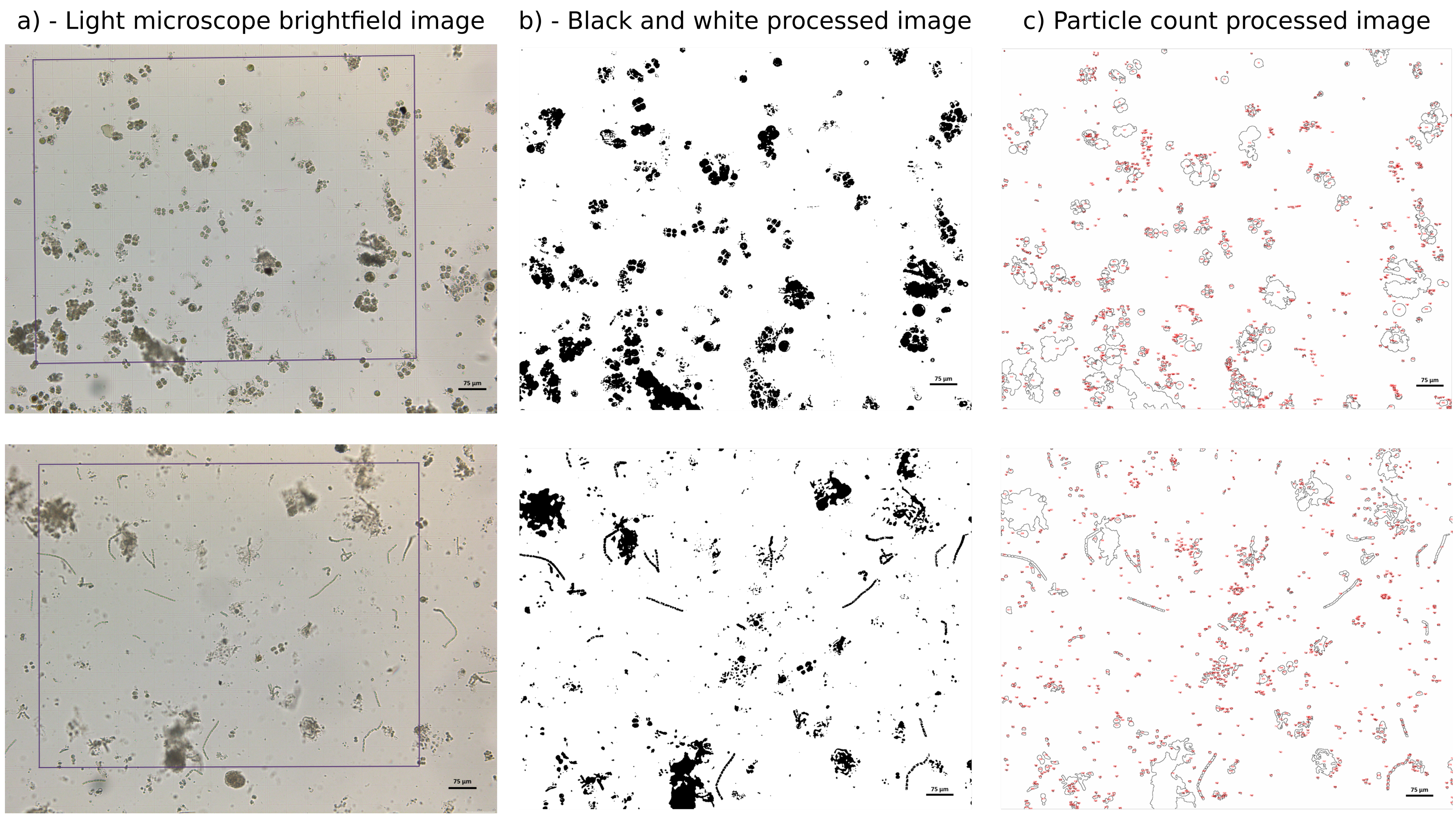

Supplementary Figure 3. **ImageJ2 brightfield image processing.** Cell density determination using microscopy and ImageJ2 software. Representative images showing detection of unicellular (single, dividing or clumped) (Rothera Point, Ryder Bay, sample 80) and filamentous algae (Rothera Point, Ryder Bay, sample 29). a) shows brightfield images. To obtain the brightfield images, melted snow samples that were preserved in 2% formaldehyde at the Bonner laboratory for analysis at Cambridge UK were mixed by gentle shaking and a 15µl sub-sample was placed directly onto the haemocytometer (Neubauer-improved) and imaged using a Leica DM600B microscope. To count the cells a 5 x 4 grid square was drawn using the haemocytometer grid lines on the brightfield image and cropped (a). b) Images were then converted to 8-bit greyscale and a threshold was applied (default, B&W) so that the cells appear black on a white background. The 'despeckle' function was used to remove background noise. c) The 'set scale' function was used by tracing the scale bar on the image to additionally determine cell size in μm^2. Cells (including any residual extracellular polymeric substances (EPS) and mineral debris) were automatically counted using the 'analyse particles' function using a size range of 0 – infinity μm^2 and circularity of 0.00 - 1.00.
